# Effectiveness of Rehabilitation Exercise in Improving Physical Function of Stroke Patients: A Systematic Review

**DOI:** 10.3390/ijerph191912739

**Published:** 2022-10-05

**Authors:** Kyung Eun Lee, Muncheong Choi, Bogja Jeoung

**Affiliations:** 1 Department Sport Industry Studies, Yonsei University, Seoul 03722, Korea; 2 Department Exercise Rehabilitation, Gachon University, Incheon 21936, Korea

**Keywords:** stroke rehabilitation, stroke exercise, stroke therapy, systematic review

## Abstract

Rehabilitation is a crucial part of recovery for stroke survivors, and numerous studies have examined various exercises and treatments of stroke. In addition, it is very important for patients to choose the timing of rehabilitation and what kind of rehabilitation they will proceed with. The purpose of the current study is to examine research investigating the effects of rehabilitation exercise programs in recovery of physical function in patients with stroke, based on aspects of their physical function, physical strength, and daily activities, and systematically examine their effects. Therefore, through systematic review, we have investigated the effects of interventions in rehabilitation exercise programs for recovery of physical function in patients with stroke. We collected relevant publications through the databases MEDLINE/PubMed and Google scholar. Twenty-one articles were ultimately selected for the analysis. We classified the rehabilitation programs and identified the trends of treatment for stroke survivors. Our review indicated that task-oriented therapy is still dominant, but various types of combined rehabilitations have been attempted. In addition, it was identified that physical and active rehabilitation were required rather than unconditional rest, even at an early stage. Home-based treatment was used for rapid recovery and adaptation to daily life during the mid-term period.

## 1. Introduction

Stroke is a cerebrovascular disease that occurs when blood supply to the brain is interrupted, or when bleeding occurs in brain tissue, resulting in loss of brain function [1]. Stroke is a terrifying disease occurring every two seconds, with people dying every six seconds due to stroke worldwide, and 15 million new cases occurring yearly. Approximately 40% of patients suffer from functional impairment after stroke onset, and 15–30% experience severe motor, sensory, cognitive, perceptual, and/or language impairments [2,3]. In particular, more than 85% of patients with stroke experience hemiplegia, which results in impaired upper limb function and decreased motor ability [4]. This impairment is a major factor that affects the ability to balance, and the levels of daily and social activities [5].

Rehabilitation is vital for minimizing sequelae after stroke, and patients who undergo continuous professional and systematic rehabilitation following the acute phase tend to recover rapidly [6,7]. Drug and rehabilitation therapy are currently practiced rehabilitation treatments for stroke. Various interventions can be applied for recovery, such as bilateral training, repetitive task training, constraint-induced movement therapy, electrical stimulation, robotic therapy, and exercise [8]. Among these, exercise is crucial because it helps patients return to activities of daily life by restoring the function of impaired muscles and improving physical function. Exercise is also essential for preventing secondary complications, as was reported in a study determining that continued exercise and physical activity after a stroke reduce the risk of recurrence of cardiovascular disease and mortality [9].

Commonly used rehabilitation exercises include those for central nerve development, passive or active exercise, progressive resistance exercise, mat exercise, and balance, postural, mobility, and gait training. According to previous studies, the approaches to rehabilitation exercises are different depending on the stage of the stroke and the types of exercise (passive, isometric, isokinetic, and isotonic) [10]. In particular, studies show that applying rehabilitation exercise early after stroke is effective [11]. However, as physical or occupational rehabilitation therapy is mainly focused on the early-onset stage, essential exercise is rarely applied in the early stages of rehabilitation. In addition, rehabilitation exercise is effective if it is utilized at the appropriate time in accordance with individual functional suitability [12].

Therefore, guidelines for each type of exercise (passive/isometric/isokinetic/isotonic) are necessary, as well as programs tailored to the individual functional levels of patients, such as the time since the injury occurrence. This customized approach will help patients with stroke recover quickly even after discharge, which will save time and cost. While there are numerous studies on restoring function in patients with stroke, systematic comparative analysis studies on the effectiveness of rehabilitation exercise interventions that consider the timing of the occurrence of impairment and the type of exercise in the clinical field are difficult to find. Therefore, a systematic analysis of studies applying rehabilitation exercises for physical function recovery in patients with stroke is necessary.

This study aims to examine research investigating the effects of rehabilitation exercise programs in recovery of physical function in patients with stroke, based on aspects of their physical function, physical strength, and daily activities, and systematically examine their effects. Based on the study findings, we will present the types and programs of exercises optimized for the phase of injury in patients with stroke.

## 2. Materials and Methods

The systematic review protocol was conducted based on the Preferred Reporting Items for Systematic reviews and Meta-Analysis (PRISMA) 2020 [13].

### 2.1. Systematic Data Resource

A systematic search of relevant publications was conducted in the following electronic databases: MEDLINE/PubMed and Google Scholar, from May 2022 to June 2022. We focused on articles published between 2012 to 2022, and English articles only were involved. The main keywords were “stroke rehabilitation”, “stroke exercise”, and “stroke therapy”, and the Boolean operator “AND/OR” was applied for additional search.

### 2.2. Eligibility and Exclusion Criteria

The eligibility criteria were established according to PICO strategy including patients, intervention, comparison, and outcome, as follows:

- Patients (P): Patients with a stroke (except where a person without disabilities acts as a person with disabilities)
- Interventions (I): Rehabilitation, exercise, and/or treatment for stroke survivors
- Comparisons (C): No rehabilitation or other intervention
- Outcomes (O): The results after intervention regarding functional improvement, pain reduction, and effectiveness of treatment

Meanwhile, the exclusion criteria included meta-analysis, reviews, letters, and proceedings. Further, the articles that did not target stroke survivors were removed. In addition, publications where intervention, results, case report, or full text were unavailable were not included for review.

### 2.3. Screening, Selection, and Exrtraction Process

This review paper selected samples based on PRISMA 2020, and three researchers were involved in the selection and extraction process. Each researcher collected publications through a search engine and conducted synthesis and exclusion. As a result, duplicated and irrelevant records were excluded in the identification stage. We identified the related publications by screening the titles and abstracts. The researchers read and assessed the full text articles, and finally selected samples suitable for the study purpose.

### 2.4. Assessment of Quality

The selected publications were evaluated using the physiotherapy evidence database (PEDro) scale to identify the methodological quality [14]. The PEDro scale is an appropriate method for rating the quality of clinical treatment or intervention which assesses 11 items, including specified eligibility criteria, random allocation, concealed allocation, groups similar at baseline, subject–therapist–assessor blinding, less than 15% drop-out, intention-to-treat analysis, between-group statistical comparisons, point measures, and variability data.

## 3. Results

### 3.1. Study Selection

**Figure 1:**
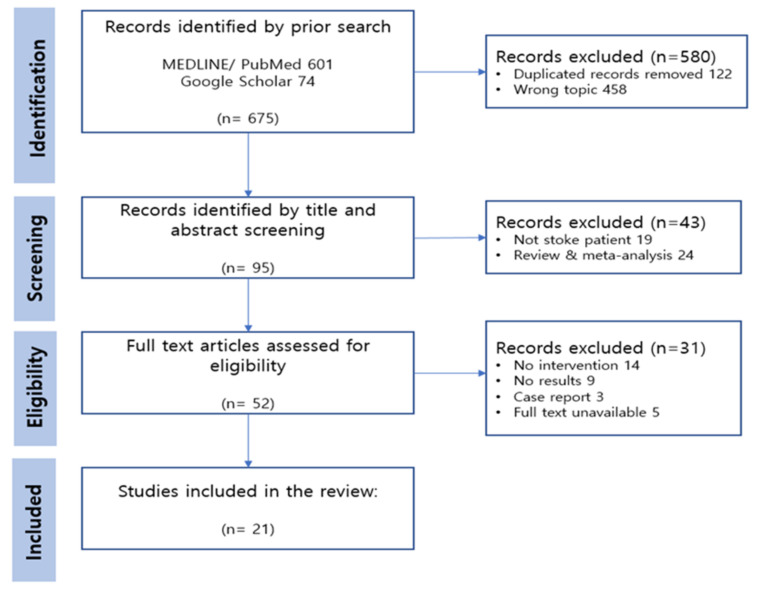
PRISMA flow diagram of the systematic review.

Through the database search, 675 articles were identified as potential publications for review. Once the titles and abstracts of the remaining 95 articles were analyzed, we retained 52 papers for assessing eligibility. Of these, 31 publications were excluded through full-text review. Subsequently, 21 articles were ultimately selected for the current review. The PRISMA flow diagram are presented in Figure 1.

### 3.2. Quality Assessment and Risk of Bias

To evaluate the quality of the selected articles, the PEDro scale was used. Thirteen of the articles in the review were of high quality (score 9–11 points), while eight demonstrated good qualities (score 6–8 points). The result of PEDro is presented in detail in Appendix A.

### 3.3. Study Characteristics

Twenty-one papers related to rehabilitation and/or exercise in stroke patients were finally analyzed, summarizing characteristics such as intervention, exercise type, control group, assessment, and results. The results can be seen in Table 1. 

The average age of participants was 60.1 years, and most publications focused on middle-aged and older adults. Most of the studies were randomized controlled trials, and three studies were of cross-over design [18,25,27]. In the case of the outcome measures, resistance and functional exercise mainly used Fugl-Meyer scale, the Wolf Motor Function Test, and Range of Motion. In terms of aerobics, the most commonly used were gait parameters and Six-Minute Walking Test.

### 3.4. Exercise Type

The exercises or rehabilitation for stroke patients were classified into resistance, aerobic, or functional (occupational). Each type was reported in detail.

In two of these studies, resistance exercise was used as an intervention [33,34]. Jung et al. [33] used an active shoulder exercise with a sling, which applied shoulder joint isometric contraction. In comparison with the control group that received bilateral arm training, researchers found the sling system decreased shoulder subluxation, and improved proprioception and upper extremity function. Kerimov et al. [34] investigated the effects of isokinetic strengthening in post-stroke patients. The participants conducted a wrist strengthening program with an isokinetic dynamometer, and the control group performed customized home-based exercises using resistance bands. The findings showed that isokinetic exercise improves motor function of upper limbs.

Five articles researched aerobic exercise [16,17,18,32,35]: three for lower extremities [16,17,18], and two for the upper body [32,35]. Stuart et al. [16]’s adaptive physical activity exercise program (APA), and Manji et al. [18]’s combined therapy showed improvement in gait speed. In addition, Ribeiro et al. [17] examined the effects of treadmill training with load. Although application of load did not indicate extra benefits, they found a minimization of weight-bearing asymmetry. In terms of upper extremity aerobic exercise, two publications used arm ergometers. Both articles reported that aerobic exercise is an effective program for stroke patients.

Functional rehabilitation was used in eight papers [19,20,22,23,24,26,28,31]. Several of them [19,23,24] implemented robot-assisted therapy, such as Bi-Manu-Track and InMotion robots. In a similar vein, two articles used electrical stimulation [22,28], and some researchers used smart devices for home care of chronic survivors [26,31]. In summary, publications researching functional therapy mainly dealt with equipment or technical treatments.

The remaining six papers applied combined rehabilitation [15,21,25,27,29,30]. Functional, resistance, and aerobic exercises were blended in these papers. Regarding combination of functional with aerobic treatment [15,30], Bovonsunthonchai et al. [15] applied both circuit class and training with motor imagery. Liner et al. [30] also conducted aerobic exercise and repetitive task practice. The results of these articles showed that there were significant therapeutic improvements in gait and walking capacity when aerobic exercise and occupational therapy were performed in parallel. In the case of functional and resistance exercises being combined [25,27,29], functional task practice was implemented with strength or power training. Of such studies, two articles [25,27] conducted isokinetic exercise in resistance training. Patten et al. [25] carried out both multi- and single-joint exercises such as lunges, squats, bicep curls (dumbbell), and supine triceps extensions (dumbbell). Corti et al. [27] also performed isokinetic movements of the upper extremity using a dynamometer. Both studies found more effective results in programs combined with resistance exercises than in single functional training. In terms of methodology especially, these publications used a cross-over design to validate the effectiveness of the treatment order. However, it was found that the order was not statistically significant. The last paper, Han et al. [29], provided stroke survivors with functional activities and strength training as an isotonic exercise. For resistance and aerobic exercise programs [21], Mazolini et al. [21] conducted two sessions for each program and found significant improvement in cardiorespiratory capacity compared with single-method therapy.

### 3.5. Exercise Type Based on the Stage of Stroke

Rehabilitation programs required by stroke patients are different according to the stage of stroke. Previous studies defined early subacute as less than three months, late subacute as three to six months, and chronic as more than six months [14]. According to this time frame, the results are as follows, except for the study of Annino (2019), without the description of the phase.

Six papers [19,20,22,29,33,35] dealt with the early subacute stage. Cecchi et al. [19] and Jong et al. [22] used electrical stimulation or robotic rehabilitation focusing on patients diagnosed with stroke 43–46 days previously. Conducting a study on patients at a similar stage, Han et al. [29] and Jung et al. [33] used resistance-combined exercises. A study by Shimodozono et al. [20] applied functional exercises for participants who were diagnosed six weeks prior. The papers reviewed relating to the early subacute phase mainly used equipment for treatment, or passive exercise.

Five papers dealt with the late acute period [17,18,27,31,32] for three to six months after occurrence. Three of them [17,18,32] conducted aerobic rehabilitation using treadmill or ergometry. Emmerson et al. [31] identified the effect of home-based exercise for stroke patients in the late acute phase. Corti et al. (2012) [27] compared resistance training and functional task practice.

The chronic stage was dealt with in nine articles [15,16,21,23,24,25,26,30,34]. Four of these used complex exercise [15,21,25,30] which is the combination of functional, aerobic, and resistance exercises. In the case of single rehabilitation, there are three articles [16,26,34] for chronic patients. Stuart et al. [14] used APA-stroke, which contains progressive exercises and coordination trainings necessary for daily activities. Chae et al. [26] studied the effect of home-based functional therapy using smart watches to help chronic patients train steadily. Further, Kerimov et al. [34] conducted isokinetic training in the paretic upper body. Hung et al. [23] and Hsieh et al. [34] both examined robot-assisted therapy, which is relevant to functional therapy.

## 4. Discussion

The goal of stroke rehabilitation is to minimize patients’ impairment and recover daily activities [36]. The therapy and training for stroke have been studied for a long time, but the results of the various interventions are too sporadic to be chosen efficiently for practical aspects. To the best of our knowledge, research regarding the classification of exercise types and exercise types according to stroke stage are still insufficient. Therefore, this study attempted to classify the effects of intervention for stroke patients through a systematic literature review based on exercise type and the phase of stroke.

### 4.1. Exercise Type

While previous research investigated the application of only single exercises, complex and/or combined exercises have recently been approached for effective rehabilitation and therapy [37]. Through the six articles investigating combined exercises, we found they were effective in improving upper limb and walking ability compared to the performance of a single exercise. Veerbeek et al. [38] also suggested that new rehabilitation access in the form of physical activity combined with novel treatments is considered very promising. In addition, regarding exercise-based rehabilitation, specifically three types of contraction movements are conducted: isometric, isokinetic, and isotonic training.

Despite these diverse programs, it can be seen from the guidelines and previous articles that task-oriented therapy is still dominant in rehabilitation for patients with stroke [39,40]. However, our outcomes and some previous studies showed that the trends of intervention-applying technologies included robot-assisted, electrical stimulation, and virtual reality [41,42,43]. It has been transformed from the passive help of therapists and simple repetitive forms to a relatively systematic rehabilitation by adopting additional methods. This can be the salient approach in stroke treatment, where continuity of participation is the most important. Moreover, the reason why tele-medication and home-based treatment related studies are increasing can be interpreted in a similar context. In addition, it was found that studies of cross-over design are being conducted in the field of stroke rehabilitation, which confirmed the importance of the effectiveness based on the order of intervention [25,27]. Although task-oriented therapies are still predominantly used for stroke survivors, various forms of combined exercise have recently been attempted.

### 4.2. Exercise Type Based on the Stage of Stroke

Prior publications regarding post stroke rehabilitation indicated that future studies should consider the optimal timing, type, and frequency of treatment [40,44]. According to our findings, in the early stages, therapy using electrical/robotic aid or passive types of exercises were mainly performed for impairment. The guidelines for adult stroke written by Winstein et al. [40] also agreed on the use of assistive technologies in early post-onset. In Kim et al.’s literature review [45], initial treatment was important to prevent complications. According to their review, there were many studies on exercises using instruments for spasticity. Therefore, it was confirmed that many studies partially supported the results of this study. Stroke survivors generally have a strong preference for rest periods over exercise-based rehabilitation [46], and to our knowledge, bed rest is considered the best solution, at least in the early stages. However, from the results of the current study, it was found that motor activities and mobilization were required from the initial phase. Askim et al. [47] agreed that the time of bed rest in the early stages of stroke was related to negative functional effects three months later. The results indicate that physical and active rehabilitation is required even during the acute stages following stroke rather than unconditional rest.

The program of late acute phase mostly consists of aerobic and home-based exercises. The second stage of rehabilitation, late acute period, requires adaptation and recovery to daily life. Similar outcomes were identified in a systematic review conducted by Kendall et al. [48], which found that continuous aerobic exercise after two to six months of stroke improved walking ability. Oliver et al. [49] also stated the beneficial effects of cardiovascular exercise in subacute stages. In terms of home-based rehabilitation, the severity of illness and comorbidities [40] should be considered. Chi et al. [50] demonstrated that stroke patients in both the acute (less than six months after stroke onset) and chronic periods (more than six months) experienced improvement in physical function. In addition, the survivors in the acute level showed greater improvement. These results are partially related to the results of the current study, in that home-based treatment is effective in the acute stage. We confirmed that the late acute period is a step for adaptation and recovery for stroke patients, and the focus here is on self-treatment and/or home-based therapy. However, further studies are required to clearly distinguish the rehabilitation based on the specific acute phase.

In the chronic stage of six months or more, our review found that combined exercise programs and tele-rehabilitation using smart devices were mainly conducted. Functional independence and autonomy may be important in this period [51]. From this point of view, computerized alert systems and mobile devices might be effective for stroke survivors in the chronic phase. However, Nam et al. [52] postulated mobile applications and smart devices are beneficial tools for treatment of stroke patients in the acute phase, which shows the opposite outcome from this study. Research related to tele-rehabilitation is increasing, but studies examining the effects of timing are insufficient. More research is therefore required regarding at which phase internet-based intervention is appropriate. Although it is not clearly distinguished by stage, it was found to be partially consistent with the guidelines in previous studies. In future studies, it is necessary to select specific rehabilitation by subdividing the stages. This study has several limitations. First, it does not include articles written in English. Previous articles on related topics written in Spanish and German might have been excluded. Second, this study did not distinguish the type of stroke, such as ischemic or hemorrhagic stroke, because it was not mentioned in the selected papers.

## 5. Conclusions

This systematic review examines the effects of interventions and types of rehabilitation based on the stroke phase. We confirmed that task-oriented therapy is still dominant, but various types of combined rehabilitations have been attempted academically. In addition, it was identified that during the initial stage, physically active rehabilitation was required rather than unconditional bed rest. In terms of the mid-term period, home-based treatment was applied for recovery and adaptation to daily life. According to this approach, we provide an overview of applicable guidelines and the specific types and programs of exercises optimized to the period of injury in patients with stroke. The patient’s stage and period after stroke onset do not completely represent the severity of their impairment. However, by establishing guidelines based on period, it will be possible to suggest post-stroke care suitable for each patient. Therefore, this study attempted to analyze the rehabilitation program for stroke survivors and provide appropriate exercises according to the patient’s stage. Future studies can consider a rehabilitation program suited to patient characteristics by further subdividing the stages.

## Figures and Tables

**Table 1:** Characteristics of the studies included in the systematic review.

Study ID	Participants (Number, Intervention, Control)	Intervention	Exercise Type	Control	Assessment	Results
Bovonsunthonchai et al. (2020) [15]	40 participants (20, 20) Age: 49.9 Time since stroke : 3–12 months	Structured Progressive Circuit Class Therapy (SPCCT) + MI (Motor imagery) Duration: 90 min Frequency: 3 times/week (4 weeks)	Functional Aerobic	SPCCT + Health Education Duration: 90 min Frequency: 3 times/week (4 weeks)	Temporo-spatial gait: FDM Strength: dynamometer Step length, time: SI	Temporo-spatial gait: + (*p* < 0.05), except for the step length of the unaffected limb (*p* = 0.063). Step length: + (*p* < 0.001) Step time: X (*p* > 0.05) Hip flexor: + (*p* = 0.002) Knee extensor: + (*p* = 0.014)
Stuart et al. (2019) [16]	76 participants (43, 33) Age: 63.9 Time since stroke : 5.1 years	APA-Strokes (progressive exercise with gait, standing and seated coordination exercises) Duration: 60 min Frequency: 3 times/week (6 weeks)	Aerobic	Sittercise (performed in a seated position) Duration: 60 min Frequency: 3 times/week (6 weeks)	Gait velocity: 6 MWT BBS (Berg balance scale), SPPB (Short Physical Performance Battery), the 30-foot timed walk, the Stroke Impact Scale (SIS)	Gait speed: + (*p* = 0.004) 30-foot walk: + (*p* = 0.02) SPPB: X (*p* = 0.54) BBS: X (*p* = 0.23) SIS: X (*p* = 0.90)
Ribeiro et al. (2020) [17]	38 participants (19, 19) Age: 57 Time since stroke : 3 months	Constraint-induced movement therapy (CIMT): treadmill training with load (5% of body weight) on the nonparetic limb Duration: 30 min Frequency: 2 times/week (9 sessions)	Aerobic	Treadmill training without load Duration: 30 min Frequency: 2 times/week (9 sessions)	Ground reaction force (GRF) from static and dynamic trials Swing time symmetry ratio	Static GRF of the paretic limb: + (*p* = 0.037) Control group’s dynamic GRF: + (*p* = 0.021) Swing time: X (*p* = 0.190)
Manji et al. (2018) [18]	30 participants (15, 15) Age: 62.2 Time since stroke : at least 4 months	Transcranial direct current stimulation (tDCS) + body weight-supported treadmill training (BWSTT) → Sham stim + body weight-supported treadmill training (BWSTT) Duration: 20 min Frequency: 7 times/week (2 sessions)	Aerobic	* Crossover design G1: tDCS + BWSTT → Sham stim + BWSTT G2: Sham stim + BWSTT → tDCS + BWSTT	Gait speed: 10 MWT Walking ability: Timed Up and Go (TUG) Lower limb: FMA-LE, TCT, POMA	G1′s Gait speed: + (*p* < 0.005) G1′s Walking ability: + (*p* < 0.005) Effect with the groups or interaction: X
Cecchi et al. (2021) [19]	224 participants (113, 111) Age: 68.5 Time since stroke : 46.5 days	Robotic rehabilitation + conventional physiotherapy (6 times/week) Duration: 45 min Frequency: 5 days/week (30sessions)	Functional (passive)	Task-oriented exercises) + Conventional physiotherapy (6 times/week)	FMA-UE	Age-FMA-UE: X (*p* = 0.603) * Age is associated with the outcome after conventional but not robotic rehabilitation.
Shimodozono et al. (2012) [20]	49rticipants (26, 23) Age: 65 Time since stroke : 6.8 weeks	Repetitive facilitative exercise (elicit movement of the shoulder, elbow, wrist, and fingers + passive stretching) + dexterity-related training (30 min) Duration: 40 min Frequency: 5 days/week (20 sessions)	Functional (passive)	Conventional upper-extremity rehabilitation program	ARAT(Action Research Arm Test) FMA	ARAT: + (*p* = 0.009) FMA: + (*p* = 0.019)
Marzolini et al. (2018) [21]	68 participants (35, 33) Age: 63.7 Time since stroke : 11.5 months	Aerobic and resistance training (AT + RT) Duration: 20–60 min Frequency:5 times/wk (6 months) (duration or intensity was increased)-2 sessions (AT) 8weeks-2 sessions (RT) (1 to 2 sets of 10 to 11 exercises)	Aerobic Resistance (isotonic)	AT	Cardiorespiratory Fitness, Body Composition and Dietary Assessment. Maximal Isometric Strength 6 MWT, Sit-to-Stand and Stair Climb Performance Exercise Logs, Adherence to Exercise, Exercise Performance, and Adverse Event Reporting	Body lean mass: + (*p* = 0.039) Predominantly trunk: + (*p* = 0.02) affected-side limbs: + (*p* = 0.04), VO2VT: + (*p* = 0.046) Muscular strength: + (*p* < 0.03) Both groups yielded similar and significant improvements: 6 MWT: X (*p* = 0.8) VO2peak: X (*p* = 0.9) Sit-to-stand time: X (*p* = 0.05), Stair climb performance: X (*p* = 0.97)
Jong et al. (2013) [22]	46 participants (23, 23) Age: 57.2 Time since stroke : 43 days	Multidisciplinary stroke rehabilitation (Cyclic neuromuscular electrical stimulation (NMES)) Duration: 45 min Frequency: 2 times/wk (16 sessions)	Functional (passive)	Sham stretch positioning procedure + simultaneous sham conventional TENS	ROM Pain in the hemiplegic shoulder: Shoulder Q	Passive range of motion: X (*p* = 0.217) No significant difference between the groups (r2 = 1.53, *p* = 0.217
Hung et al. (2019) [23]	68 participants (20, 10) Age: 55.54 Time since stroke : 23 months	Robot-assisted therapy (RT) BMT robot vs. IMT robot Duration: 90–100 min Frequency: 5 times/wk (20 sessions)	Functional (passive)	Individualized occupational therapy	FMA-UE Muscle spasticity: MAS Quality of movement: MAL Muscle strength of the affected arm: MRC	FMA-UE: IMT > BMT (*p* < 0.01) MAS: IMT + (*p* = 0.01), BMT X (*p* = 0.55) CT X (*p* = 0.44) MAL: IMT + (*p* = 0.01) BMT X (*p* = 0.55) CT X (*p* = 0.44) MRC: IMT X (*p* = 0.27) BMT + (*p* = 0.01) CT: X (*p* = 0.3)
Hsieh et al. (2018) [24]	44 participants (32, 12) Age: 54 Time since stroke : 21 months	Robot-assisted therapy (RT) P-IMT vs. D-IMT Duration: 90–100 min Frequency: 5 times/wk (20 sessions)	Functional (passive)	Conventional rehabilitation +FTP	FMA-UE Muscle spasticity: MAS Quality of movement: MAL Muscle strength of the affected arm: MRC Wrist-worn accelerometers	Total MRC: D-IMT > P-IMT, CT (*p* = 0.04, *p* = 0.04) FMA:X (*p* = 0.77), proximal FMA: X (*p* = 0.97), proximal MRC: X (*p* = 0.12) * Distal upper-limb robotic rehabilitation using the D-IMT had superior effects on distal muscle strengthen
Patten et al. (2013) [25]	19 participants (9, 10) Age: 68 Time since stroke : 12 months	HYBRID (combined Functional Training Practice + Power training) Duration: 75 min Frequency: 5 times/wk (24 sessions)	Resistance (isokinetic) Functional	* Crossover design G1: FTP→HYBRID G2: HYBRID→FTP	FMA-UE the Ashworth Scale WMFT-FAS Functional Independence Measure: FIM	WMFT-FAS: HYBRID > FTP (*p* < 0.05) Treatment order: X (*p* = 0.43) FMA: X (*p* > 0.05) FIM: + (HYB > FTP, *p* < 0.05) Ashworth score: X (*p* > 0.05)
Chae et al. (2020) [26]	23 participants (17, 6) Age: 61.4 Time since stroke : at least 6 months	Smart watch based Home-based rehabilitation Duration: 30 min Frequency: 12 weeks	Functional	Tele-rehabilitation service	FMA-UE WMFT-FAS Grip power ROM BDI: Beck Depression Inventory	WMAFT: + (*p* = 0.02) Grip power: X (*p* = 0.46) FMA-UE: X (*p* = 0.34) ROM: flexion: + (*p* < 0.001) Extension: X (*p* = 0.16) Internal rotation: + (*p* = 0.001) External rotation: X (*p* = 0.2)
Corti et al. (2012) [27]	14 participants (14) Age: 59.8 Time since stroke : 15 weeks	Dynamic resistance training (POWER) vs. Functional task practice (FTP) Duration: 90 min Frequency: 3 days/wk (30 sessions)	Resistance (isokinetic) Functional	* Crossover design (10 week+10 week) Order1: FTP→POWER Order2: POWER→FTP	UEFMMS the Ashworth Scale European Stroke Scale CMHAI Kinematics of functional reach to grasp	Treatment effect (FTP vs. POWER): X (both groups improved without differential treatment effects) Treatment order: X (*p* > 0.05) Period effect: X (*p* > 0.05) Kinematic: Treatment effect: POWER > FTP Treatment order: G2 > G1
Annino et al. (2019) [28]	37 participants (19, 18) Age: 68.6 Time since stroke : null	Supervised physical therapy + Segmental muscle vibration (SPT-SMV) Duration: 30 min Frequency: 3 days/wk (24 sessions)	Functional (passive)	Supervised physical therapy (SPT)	Barthel index (BI) the Ashworth Scale (MAS) Manual muscle testing (MMT) ROM	Both groups improved in BI, Elbow ROM, Elbow muscles strength Muscle tone in elbow joint improved only in SPT-SMV (*p* = 0.008)
Han et al. (2012) [29]	32 participants (11, 10, 11) Age: 50.2 Time since stroke : 38–42 days	Different intensities of arm rehabilitation training (correct positioning and carrying of the arm; passive, assisted and active movements; strength training; practice of functional activities) Duration: G1: 1 h/G2: 2 h/G3: 3 h Frequency: 5 days/wk (30 sessions)	Resistance (isotonic) Functional (passive)	1 h (group A) 2 h (group B) 3 h (group C)	FMA-UE ARAT (Action Research Arm Test) Barthel index (BI)	FMA and ARAT: Group C > A, B (*p* < 0.05) BI: X (*p* > 0.05).
Linder et al. (2020) [30]	43 participants (16, 14, 13) Age: 56 Time since stroke : 13 months	G1: Forced aerobic exercise (60% to 80% of their heartrate reserve) + repetitive task practice (FE + RTP) G2: Voluntary aerobic exercise + RTP (VE + RTP) Duration: 90 min Frequency: 3 times/wk (24 sessions)	Aerobic Functional (passive)	G3: RTP only	6 MWT	6 MWT: G1: + (*p* < 0.001) G2: + (*p* < 0.001) G3: X (*p* = 0.21)
Emmerson et al. (2017) [31]	62 participants (30, 32) Age: 66 Time since stroke : 4 months	Home exercise video on smart technology and automated reminders (stretching, strengthening, fine motor/coordination) Duration & Frequency depended on the participants (average 38 min/day)	Functional (passive)	Paper-based home exercise program	Adherence WMFT Satisfaction	Adherence: X (*p* > 0.05) WMFT: X (*p* > 0.05) Satisfaction: X (*p* > 0.05) * smart technology was not superior to standard paper-based
Topcuoglu et al. (2015) [32]	40 participants (20, 20) Age: 65.95 Time since stroke : 3.5 months	Upper extremity aerobic exercise (UEAE) (arm crank ergometry) Duration: 30 min Frequency: 5 days/wk (20 sessions)	Aerobic	Conventional physiotherapy	CPRS clinical determinants Functional independence measure (FIM) Nottingham Health Profile (NHP) Beck Depression Scale scores (BDS)	FIM sub scores (motor and cognitive): + (*p* > 0.05) NHP: + (*p* > 0.005) BDS: + (*p* = 0.005) Clinical determinants: significant pain relief and decline in signs and symptom
Jung et al. (2019) [33]	36 participants (18, 18) Age: 58.5 Time since stroke : 28.65 days	Active shoulder exercise with a sling suspension system Duration: 40 min Frequency: 5 days/wk (20 sessions)	Resistance (isometric)	Bilateral arm training	Shoulder subluxation distance Shoulder proprioception FMA-UE the manual function test (MFT)	Subluxation: + (*p* = 0.001) Proprioception: + (*p* = 0.046) FMA: + (*p* = 0.002) MFT: + (*p* = 0.007)
Kerimov et al. (2021) [34]	24 participants (12, 12) Age: 54.3 Time since stroke : at least 6 months	Isokinetic training in paretic upper extremity Duration: 40 min Frequency: 3 days/wk (12 sessions)	Resistance (isokinetic)	Tailored strengthening exercises with exercise bands	Isokinetic peak torque FMA-UE Stroke Impact Scale (SIS) Disabilities of the Arm, Shoulder and Hand (DASH) questionnaire Grip strength Peak isometric strength	SIS: isokinetic group had higher scores on nearly every domain Extensor peak torque at 60°: + (*p* = 0.007) Extensor peak isometric muscle strength: + (*p* = 0.007) DASH after 4weeks after the end of treatment: + (*p* = 0.014) Grip strength: X (*p* > 0.05)
Pinheiro et al. (2021) [35]	20 participants (10, 10) Age: 66.2 Time since stroke : null (Acute)	Upper limb cycle ergometer (ULCE) Duration: 20 min Frequency: 5 days/wk (20 sessions)	Aerobic	Conventional physiotherapy	Upper limb strength and grip Trunk impairment scale (TIS) Level of independence: Modified Rankin scale (MRS)	ULCE: all variables showed Superior (*p* = 0.005) TIS: + (*p* < 0.001) MRS: + (*p* < 0.001)

## Data Availability

Not applicable.

## Appendix A

**Table A1:** Assessment of Methodological quality by PEDro scale.

Selected Paper	1	2	3	4	5	6	7	8	9	10	11	Score
1	Yes	Yes	Yes	Yes	Yes	No	Yes	Yes	Yes	Yes	Yes	10
2	Yes	Yes	Yes	Yes	Yes	No	No	No	Yes	Yes	Yes	8
3	Yes	Yes	No	Yes	Yes	Yes	Yes	Yes	Yes	Yes	Yes	10
4	Yes	Yes	No	Yes	No	No	Yes	Yes	Yes	Yes	Yes	8
5	Yes	No	No	Yes	No	No	No	Yes	Yes	Yes	Yes	6
6	Yes	Yes	Yes	Yes	Yes	Yes	Yes	Yes	Yes	Yes	Yes	11
7	Yes	Yes	Yes	Yes	Yes	Yes	Yes	Yes	Yes	Yes	Yes	11
8	Yes	Yes	Yes	Yes	Yes	Yes	Yes	Yes	Yes	Yes	Yes	11
9	Yes	Yes	No	Yes	Yes	No	Yes	Yes	Yes	Yes	Yes	9
10	Yes	No	No	Yes	Yes	Yes	Yes	Yes	Yes	Yes	Yes	9
11	Yes	Yes	Yes	Yes	Yes	Yes	Yes	Yes	Yes	Yes	Yes	11
12	Yes	Yes	No	Yes	No	No	No	Yes	Yes	Yes	Yes	7
13	Yes	Yes	No	Yes	Yes	Yes	Yes	Yes	No	Yes	Yes	10
14	Yes	Yes	Yes	Yes	Yes	No	No	Yes	Yes	No	Yes	7
15	Yes	Yes	No	No	No	Yes	No	Yes	Yes	Yes	Yes	6
16	Yes	Yes	Yes	Yes	Yes	No	No	Yes	Yes	No	Yes	8
17	Yes	Yes	Yes	Yes	Yes	Yes	Yes	Yes	Yes	Yes	Yes	11
18	Yes	Yes	Yes	Yes	Yes	No	Yes	Yes	Yes	Yes	Yes	11
19	Yes	Yes	Yes	Yes	Yes	Yes	Yes	Yes	Yes	Yes	Yes	11
20	Yes	Yes	No	Yes	No	No	No	Yes	Yes	Yes	Yes	8
21	Yes	Yes	Yes	Yes	Yes	Yes	Yes	Yes	Yes	No	Yes	10
